# Lead intoxication in dogs: risk assessment of feeding dogs trimmings of lead-shot game

**DOI:** 10.1186/s12917-016-0771-z

**Published:** 2016-07-25

**Authors:** Helga R. Høgåsen, Robin Ørnsrud, Helle K. Knutsen, Aksel Bernhoft

**Affiliations:** 1 Norwegian Veterinary Institute, P.O. box 750, Sentrum, NO-0106 Oslo Norway; 2 National Institute of Nutrition and Seafood Research (NIFES), P.O. box 2029, Nordnes, 5817 Bergen Norway; 3 Norwegian Institute of Public Health, P.O. box 4404, Nydalen, 0403 Oslo Norway

**Keywords:** Lead toxicity, Dogs, Hunting, Ammunition, Review

## Abstract

**Background:**

Expanding lead-based bullets, commonly used for hunting of big game, produce a scattering of lead particles in the carcass around the wound channel. Trimmings around this channel, which are sometimes fed to dogs, may contain lead particles. The aim of this study was to assess potential health effects of feeding dogs such trimmings.

**Results:**

Lead ingestion most commonly causes gastrointestinal and neurological clinical signs, although renal, skeletal, haematological, cardiovascular and biochemical effects have also been reported. Experimental data indicate that a daily dose of around 1 mg lead as lead acetate/kg body weight for ten days may be considered as a Lowest Observed Effect Level in dogs. Acute toxicity documentation from the Centers for Disease Control and Prevention indicates 300 mg/kg body weight as the lowest dose of lead acetate causing death in dogs after oral ingestion. Our assessment suggests that dogs fed trimmings of lead-shot game may be affected by the amounts of lead present, and that even deadly exposure could occasionally occur. The intestinal absorption of lead from bullets was assumed to be 10–80 % of that of lead acetate, reflecting both the variability in particle size and uncertainty about the bioavailability of metallic lead in dogs.

**Conclusions:**

Despite data gaps, this study indicates that feeding dogs trimmings of lead-shot game may represent a risk of lead intoxication. More research is needed to assess the exact consequences, if lead-based bullets are still to be used. Meanwhile, we recommend that trimmings close to the wound channel should be made inaccessible to dogs, as well as to other domestic or wild animals.

## Background

Hunting is a popular leisure activity in many countries. In big game hunting, lead-based expanding bullets are commonly used. Such bullets produce a scattering of lead particles in the carcass around the wound channel. The fragmentation of the bullet depends on the rifle calibre, bullet type, velocity at impact, distance and angle of the shot, and the possible encountering of bones, but frequently leads to lead contamination of the meat [1–3]. Lead contaminated meat represents a route of exposure for humans with high game meat consumption. Indeed, a positive correlation between game consumption and blood lead concentrations has been shown in humans [4–6]. Several reports and risk assessments have resulted in recommendations that vulnerable groups such as children and pregnant women should limit their intake of game meat [7–10].

Many scavenging and predatory birds and mammals accidentally ingest lead gunshot or bullets/fragments if they eat unretrieved quarry or remnants discarded by hunters, and lead poisoning from ammunition sources is a well-established cause of mortality among such birds globally [5]. The hunting dog has a central role in several modes of hunting, including hunting of big game. The dogs are sometimes rewarded with a share of the prey, commonly parts that are not used for human consumption such as trimmings of meat and organs around the wound channel. In a Norwegian study, two out of 23 (9 %) hunting team leaders reported the trimmings were fed to dogs [10]. However, the potential risk for companion animals receiving such trimmings has not received attention.

The aim of the present study is was to estimate the potential health risk for dogs ingesting lead fragments in trimmings close to the wound channel from big game killed with lead-based ammunition.

## Methods

The four stages of risk assessment, comprising hazard identification, hazard characterisation, exposure assessment, and risk characterisation [11] were undertaken by help of literature search and original predictions of exposure. A Lowest Observed Effect Level (LOEL) in dogs was determined based on published literature, which was reviewed in the hazard characterisation.

### Literature search

A search strategy combining the keywords “lead-poisoning”, “lead toxicosis”, “lead bioavailability”, “dogs”, “domestic animals”, “companion animals”, “venison”, “deer”, “elk”, “moose” and “game meat” was used. A similar search using the same terms, but with «Pb» instead of «lead» was also performed. This search strategy was conducted in PubMed, Web of Science (including the Science and Social Sciences Citation Index), and Google Scholar. In addition, hand-search was used. For articles with potential relevance based on title and abstract, the full text was obtained and assessed.

### Exposure predictions

Due to the lack of optimal data in the literature, we used available data with two different approaches. Exposure was first assessed using a simple deterministic approach. A second approach was then used with a different source of data, adding Monte-Carlo simulation to account for uncertainty and variability. The model was built in Microsoft Excel 2010, with ModelRisk Professional (Vose Software 2011) as an add-in. 10 000 iterations were used. Inputs and formulas are explained and discussed in the exposure assessment.

## Results and discussion

### Hazard identification - The toxicity of metallic lead in dogs

Lead is a toxic heavy metal that has negative impacts on the gastrointestinal, nervous, renal, cardiovascular and haematological systems. The general mechanism of action is linked to lead’s affinity to proteins, e.g., thiol groups, and to its ability to substitute for calcium. Moreover, lead may influence the homeostasis of other minerals such as magnesium and zinc. Lead affects neuronal tissue causing cell death and disturbed transfer of nerve signals. The detailed mechanisms of toxicity have not yet been fully uncovered but a comprehensive review of the current knowledge of lead toxicity has been published by the European Food Safety Authority [8]. Lead poisoning in dogs most often occurs as a result of oral exposure to lead through contaminated water, lead-containing paint, or other lead-containing items [12]. Lead poisoning due to ingestion of lead-containing particles has been reported on multiple occasions for dogs, particularly young dogs with aberrant eating habits [12]. Most experimental data on effects of lead exposure are on lead acetate, while dogs may in many cases, as with meat from lead-shot game, ingest metallic lead.

### Hazard characterisation – Bioavailability, distribution and dose–response

#### Bioavailability of metallic lead to dogs

There is little information on the bioavailability of lead to dogs. Absorption of lead depends on many factors such as chemical form, particle size, age and nutrition of the animal [13–16]. In the rat, the absorption of metallic lead has been shown to be 60–80 % of that of lead acetate, under conditions of comparable particle sizes (<53 μm diameter), and reduced with increasing particle size, due to reduced surface-area-to-mass ratio [14]. Lead particles in the nanometre range can be directly absorbed by pinocytosis in the rat duodenum [17]. Elevated blood concentrations of lead were found in experimental pigs fed with meat from lead-hunted roe deer [3], and the association between human blood lead concentrations and the consumption of lead-hunted game meat suggests significant bioavailability of metallic lead [4–6, 18, 19]. Furthermore, larger lead fragments residing in gastrointestinal system may result in toxicity [20, 21]. Radio-opaque objects in the gastrointestinal tract of companion animals are frequently reported in incidences of lead poisoning [22]. Accidental ingestion of ammunition accounted for 3.3 % and 17.6 % of dog lead poisoning cases in a study comparing the US and France, respectively [12]. The relatively low pH of the gastric juice of dogs may lead to higher absorption and bioavailability of lead in dogs compared with other animals such as rats, pigs and humans.

#### Distribution and elimination of lead in dogs

Once absorbed, lead is readily distributed by transport in red blood cells, where it is bound to haemoglobin, or attached to other blood proteins such as albumin. Lead crosses the blood–brain and placental barriers, and the distribution of lead in different body compartments of dogs at equilibrium shows preferential accumulation in bone > > liver > kidney > spleen > pancreas > blood > brain [23, 24]. The residence time of lead is rapid in blood compared to bone and suggests different elimination rates in different body compartments [25]. The biological half-life for lead in bone has been estimated to 346 days for dogs [26]. The elimination of lead is mainly through biliary clearance to faeces; Lloyd et al. [23] showed that 75 % of intravenously injected radiolabelled lead (^210^Pb) in beagle dogs could be found in the faeces.

#### Clinical signs, physiopathology and dose–response of lead poisoning in dogs

Blood lead concentrations above 400 μg/L can be considered as a marker of lead poisoning in dogs. [22] However, blood lead concentrations are not necessarily correlated with severity of the poisoning [20, 27]. Gastrointestinal and neurological symptoms are the most common signs of lead poisoning, with colic and agitation as clinical manifestation. Berny et al. [12] reported that gastrointestinal disorders were more frequent than neurological disorders, the latter being more frequent in younger (< 5 years old) than in older dogs. Table 1 summarises reported clinical and pathological findings in lead-exposed dogs.

**Table 1:** Reported effects of lead poisoning in dogs

Effect category	Clinical and pathological findings	References
Gastrointestinal	Vomiting, diarrhoea, abdominal pain (“lead colic”), delayed gastric emptying	[12, 21, 22]
Neurological	Tremor, spasms, epileptic seizures, agitation, lethargy, ataxia, anorexia, cortical neuronal necrosis	[12, 22, 28]
Renal	Proximal tubular epithelial cell damage and necrosis, enlarged kidneys	[24, 28]
Skeletal	Sclerosis, delayed closure of vertebral epiphyses, lead lines	[24, 28]
Haematological	Anemia (reduced erythrocyte, lowered haemoglobin, elevated mean cell volume), basophilic stippling, nucleated erythrocytes, elevated leukocyte count	[21, 22, 28]
Cardiovascular	Hypertension, endothelial degeneration and capillary proliferation in the brain	[28, 31]
Biochemical	Reduced ALA-D activity, increased urinary ALA, hypoproteinemia	[20–22, 24, 31]

Although gastrointestinal distress is a common sign of lead poisoning, lesions in the epithelium are generally not found in the gastrointestinal tract. A low frequency of gastro-oesophageal ulcers have been reported but can not fully explain the gastrointestinal symptoms [28].

Degeneration and necrosis of cortical neurons were prevalent in dogs with neurologic disorders as reported by Zook [28]. The occipital and parietal lobes were the main sites of lesions but lesions were also found in cerebellar Purkinje cells and in the hippocampus. Endothelial degeneration and capillary proliferation were observed in dogs with neurological symptoms lasting more than eight days. Furthermore, swollen astrocytes, thickened meninges and oedematous separation of connective tissue fibres were observed in dogs with a prolonged course of nervous lead intoxication.

The adverse effects of lead on the haematological system are mainly the result of its perturbation of the heme biosynthesis pathway. The activity of aminolevulinic acid dehydratase (ALA-D), an enzyme in the heme synthesis pathway, is negatively correlated with lead. Consequently, the conversion of delta-aminolevulinic acid (ALA) to porphobilinogen by ALA-D is disturbed leading to elevated concentrations in blood and urine of ALA in lead poisoned dogs [20, 29]. Penumarthy et al. [30] fed 0 mg (control), 2 mg and 5 mg lead as lead acetate/kg bw/day to two-month old beagles for 13 weeks. Reduced body weight and ALA-D activity as well as elevated nucleated erythrocyte count, erythrocyte protoporphyrins and urinary ALA were observed in dogs exposed to lead at both treatment levels.

The classic effects of lead in the kidneys are characterised by proximal tubular nephropathy, glomerular sclerosis, interstitial fibrosis and related functional deficits, including proteinuria, impaired transport of organic anions and glucose, and depressed glomerular filtration rate. The proximal tubuli epithelium seems to be the main site of lesions which affect both nuclei and cell morphology [28]. Stowe et al. [24] fed a calcium and phosphorus deficient diet to littermate mongrel dogs from 6 to 18 weeks of age at 0 or 100 mg lead acetate/kg diet. The estimated average lead dose was around 3.3 mg lead/kg bw/day. The major pathological findings of lead poisoning were increased weight of liver, kidney and brain with histopathological lesions in liver, kidney and bones. Furthermore, hypoproteinemia and moderate electrolyte and enzyme alterations in blood were found. These relatively strong effects may be explained by the low dietary intake of calcium since this enhances the susceptibility to lead intoxication [15].

As in humans [8], cardiovascular effects may be seen in dogs after lead exposure. Fine et al. [31] showed that a daily oral dose of 1 mg lead acetate/kg bw to dogs from three months of age led to hypertension after 10 days of treatment. This elevation in blood pressure was sustained at approximately 10 % above that of paired control animals throughout the study. This hypertension was associated with a small increase in the activity of the renin-angiotensin system. However, there were no effects on extracellular fluid volumes, glomerular filtration rate or renal plasma flow indicating no renal damage or alterations in renal function. A mild state of lead poisoning was indicated by blood lead concentrations ranging from 250 to 400 μg/L as well as decreased ALA-D activity. Similarly, Mouw et al. [32] showed elevated plasma renin activity and increased urinary excretion of sodium, potassium, calcium and water due to reduced renal reabsorption of these electrolytes in dogs given 3 mg lead acetate/kg bw intravenously as a single dose.

Table 2 summarises the daily dose, duration of lead exposure and clinical findings in dogs fed dietary lead experimentally.

**Table 2:** Daily dose, duration of lead exposure and clinical findings in dogs given dietary lead experimentally

Daily dose and chemical form	Duration	Age and breed (number of dogs)	Clinical and pathological findings	References
1 mg lead acetate/kg bw/day	20 weeks	3 months old hounds (*n* = 6)	Increased blood pressure and plasma renin activity	[31]
2 or 5 mg lead acetate/kg bw/day	13 weeks	2 months old beagles (*n* = 4)	Lowered ALA-D activity, increased number of nucleated erythrocytes	[30]
50 or 100 mg lead carbonate/kg bw/day	1 week	One year old beagles (*n* = 2)	Increased hepatic enzyme activity	[38]
50 mg lead carbonate/kg bw/day	5 weeks	One year old beagles (*n* = 2)	Hepatic and renal histological changes, altered hepatic enzyme activity	[38]
~3 mg lead acetate/kg bw/day^a^	12 weeks	1 month old mongrels (*n* = 3)	Anemia, cachexia, increased organ weights, hepatic and renal lesions, bone malformation, altered blood chemistry	[24]

^a^Low Ca and P diet

In sum, the lead dose leading to adverse effects in dogs varies but a dose at 1 mg lead as lead acetate/kg bw/day can be considered as a Lowest Observed Effect Level (LOEL), with increased blood pressure observed already after 10 days [31]. According to the Centers for Disease Control and Prevention, it has been suggested that the lowest dose of lead acetate causing acute death in dogs after oral ingestion is 300 mg/kg bw [33].

### Exposure assessment - Exposure of dogs to lead through trimmings of lead-shot game

There are no data available in the literature concerning the quantity of trimmings from lead-shot game fed to dogs, nor about the lead concentrations in trimmings. However, available data may be used to estimate the lead exposure of dogs through such products, by two different approaches described below. The first approach we used was a deterministic approach based on the maximal meat intake of dogs and the maximal lead concentrations found in meat meant for human consumption (reviewed in Table 3). The second was a probabilistic approach based on residues from lead bullets in moose (results reported in Table 4). In both cases, the exposure to metallic lead was converted into exposure to lead acetate equivalents. The bioavailability of metallic lead has been shown to be 60–80 % of that of lead acetate in rats [14], and may be higher in dogs due to more acidic gastric fluid. However, the size of particles influences the bioavailability, as larger particles have lower surface area relative to weight, and may be less dissolved in the gastrointestinal tract. Hundreds of fragments radiographically counted in deer shot with lead bullets, were shown to weigh only 0.1-1.0 mg, and a considerable number were supposed to be missed due to their even smaller size [34]. We considered a reasonable range for the relative bioavailability of lead to be 10 - 80 % of that of lead acetate, accounting both for the variability in particle size of metallic lead in meat and uncertainty about the bioavailability of metallic lead in dogs.

#### Deterministic approach based on known lead concentration in meat

The normal daily feed intake of active dogs is 1.5–3 % dry matter related to their body weight [35]. If half of this is covered by fresh meat, which has a dry matter ratio at approximately 1/3, it represents about 22.5 - 45 g fresh meat/kg bw. A reasonable worst case assumption of daily intake of trimmings of lead-shot game can therefore be set at 45 g meat/kg bw. The concentration of lead in meat trimmings from the bullet channel is not known, but must be higher than concentrations reported in big game meat meant for human consumption. More bullet fragments are found closer to the wound channel, so trimmings can be expected to contain more lead [36]. Table 3 shows an overview of some reported concentrations of lead in game meat for human consumption after trimming. The results show highly varying lead concentrations, with observed maximum concentrations close to 900 mg/kg wet weight. This very high concentration is likely to have been measured close to the wound channel.

**Table 3:** Mean and maximum concentrations of lead (mg/kg wet weight) found in samples of meat from various categories of hunted animals, meant for human consumption

Source	No. of samples	Mean concentration (mg/kg w.w.)	Maximum concentration (mg/kg w.w.)	Reference
Minced moose meat	52	5.6	110	[39]
Minced moose meat	54	0.9	31	[40]
Minced venison meat	57	9.1	235	[41]
Game meat (reindeer, deer, wild pheasant)	2521	3.2	867	[8]
Wild boar	966	1.1	n.a.	[9]
Venison meat	733	0.05	n.a.	[9]
Elk meat	47	0.02	n.a.	[9]
Reindeer	490	0.06	n.a.	[9]
Hare meat	149	0.16	n.a.	[9]
Red deer	61	0.33	4.6	[42]
Wild boar	64	1.3	10.4	[42]
Red deer	82	0.22	1.5	[43]

A reasonable assumption is therefore that meat trimmings fed to dogs may commonly have lead concentrations of 900 mg/kg or more. A daily meat intake by the dogs of 45 g/kg bw corresponds therefore to a daily intake of metallic lead of approximately 40 mg/kg bw or more. Given a relative bioavailability in the range of 10 – 80 % of that of lead acetate, 40 mg/kg bw would correspond to 4 – 32 mg/kg bw per day of lead acetate equivalents.

#### Probabilistic approach based on known lead quantities released from bullets

The second approach was based on observed lead residues in moose shot in Scandinavia, as published by Stokke et al. [37]. These authors weighed the bullets before and after impact, and calculated the lead loss. Results were grouped per bullet type, and main statistics are reported in Table 4.

**Table 4:** Lead loss (in g) per bullet during moose shots

Type (i)	Name	Mean	n	SD	Min	Max
1	458 winchester magnum	7.95	7	2.36	1.39	20.73
2	9.3 × 57	3.99	6	1.79	0.04	11.68
3	375 h&h magnum	3.82	7	1.14	0.74	8.68
4	338 winchester magnum	3.72	12	0.44	1.66	6.43
5	9.3 × 62	3.54	62	0.31	0	9.82
6	300 winchester magnum	3.22	6	0.62	0.89	5.24
7	7.62 × 53r	2.96	44	0.16	0.01	5.18
8	30–06 sprg	2.93	229	0.11	0.12	10.99
9	308 norma magnum	2.86	18	0.3	0.2	4.61
10	8 × 57JS	2.84	19	0.38	0.13	5.47
11	308 winchester	2.57	446	0.06	0.01	8.86
12	7 mm remington magnum	1.75	6	0.53	0.19	3.07
13	45 / 70	1.72	14	0.35	0.06	4.8
14	6.5 × 55 mauser	1.52	44	0.17	0	5.28

From [43]. Authorisation to reproduce data granted by the journal

We included the observed variability by modelling total residues for each bullet type, by a Normal distribution with mean and standard deviation based on Stokke et al. [37], truncated at the minimum and maximum values observed. In the General scenario, the likelihood of each bullet type was based on the number of projectiles examined by Stokke et al. [37]. In addition, we considered exposure specifically with the worst case projectile (worst scenario) – the one with highest average lead loss (458 Winchester Magnum), and the best case projectile (best scenario) - the one with lowest average lead loss (6,5x55 Mauser).

There are no publications regarding the volume of meat contaminated, nor the volume of meat fed to dogs. We can still estimate the exposure of dogs fed all contaminated meat from one bullet. This may overestimate exposure since we know that some of the lead remains in the meat for human consumption (see above), but may also underestimate exposure since several bullets may be used. In the study by Stokke et al. [37], 32 % of animals were killed by more than one bullet, and an average of 1.4 shots were used per animal. These two biases may therefore reasonably compensate each other. However, if dogs are fed trimmings from several wound channels, their real exposure may be significantly higher than predictions from our model. Up to nine bullets were used in the study by Stokke et al. [37]. Multiplying the predictions of our study with the number of wound channels trimmed and fed to the dog, provide an easy way to estimate such exposure. The dog weight was assumed to be 15–25 kg, based on the most common breed used in moose hunt in Norway (Norsk elghund; Norwegian elkhound). Model inputs and formulas are summarised in Table 5.

**Table 5:** Inputs and formulas used in the probabilistic exposure assessment

Variable	Symbol	Unit	Value/Formula/Distribution	Source
Bullet type (General scenario)	i		= Discrete (Bullet (1,…14); n (1,…14))	Relates to Table 3
Bullet type (Worst scenario)	i		1	Relates to Table 3
Bullet type (Best scenario)	i		14	Relates to Table 3
Total lead in moose	Q	mg	= Normal (Mean, SD, bounds(Min, Max)) for Bullet type	Relates to Table 3
Relative bioavailability (vs. lead acetate)	k		= Uniform (10 %;80 %)	Authors, based on [14]
Exposure dose (lead acetate equivalent)	Da	mg	= k x Q	
Dog body weight	bw	kg	= Uniform(15;25)	Norsk Elghund (Wikipedia)
Exposure / kg	Da/kg	mg/kg	= Da / bw	

**Fig. 1:**
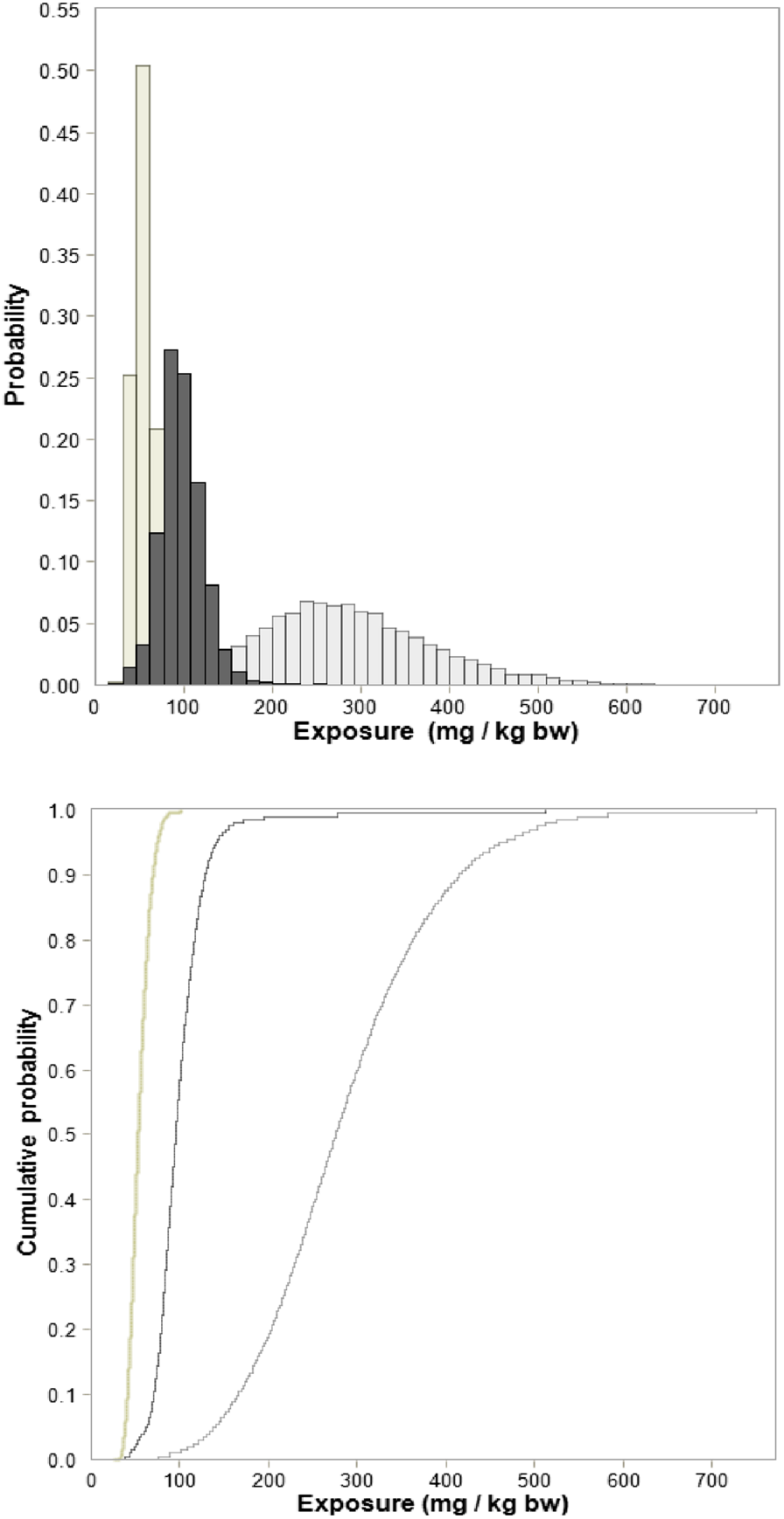
Histograms and cumulative curves of the predicted exposure to lead acetate equivalents (mg/kg bw) in hunting dogs fed the total amount of lead residues from one bullet in lead-shot big game, according to General scenario (*dark grey bars or line*), Worst scenario (*light grey bars or line*) and Best scenario (*beige bars or line*)

We predicted the total exposure of dogs to lead acetate equivalents by kg body weight. Results for the three probabilistic scenarios (general, worst and best) are summarised in Table 6 and Fig. 1.

**Table 6:** Predicted lead exposure (mg/kg bw) of dogs fed meat with the lead residues from one bullet

	General scenario	Worst scenario	Best scenario
Mean	99	284	54
St. Deviation	31	98	11
Minimum	8	43	25
Maximum	512	749	101
Median	95	275	53
95 percentile	140	460	74
99 percentile	194	549	83

Results are shown as lead acetate equivalents per body weight (mg/kg bw). The General scenario is based on a mixture of bullet types, the Worst scenario on the bullet type with highest lead loss and the Best scenario on the bullet type with lowest lead loss, according to Stokke et al. [37]

### Risk characterisation - Consequences of feeding dogs trimmings of lead-shot game

In the first, deterministic, exposure assessment, we showed that hunting dogs could be exposed to lead acetate equivalent doses above 4 - 32 mg/kg bw per day, depending on the bioavailability of metallic lead, since trimmings may have lead concentrations above those found in meat intended for human consumption. In the second, probabilistic, exposure assessment, the predicted median exposure of a dog fed the entire load of lead residue from one bullet, ranged from 53 mg/kg bw for the bullet type with lowest average residue left (6,5x55 Mauser), to 275 mg/kg bw for the one leaving most residues (458 Winchester Magnum). Maximal predicted exposure was 749 mg/kg bw. Since the lowest dose of lead acetate causing death in dogs after oral exposure is suggested to be 300 mg/kg bw [33], it can’not be excluded that some dogs may die from such an exposure.

The health effect of the lead exposure will be influenced by how long period the dogs are fed the amounts of lead. For an elkhound eating 0.7–1.1 kg meat per day (0.045 kg meat/kg bw per day), we suggest that the most heavily lead contaminated trimmings are ingested within 10–14 days. In a Norwegian study, the majority of 23 hunting team leaders reported they cut the meat privately, and most reported they removed 10–20 cm around the wound channel [10]. Personal communications from hunters to the authors also indicate that 5–10 kg meat is common to remove. The comparison with the LOEL of 1 mg lead/kg bw per day which produced increased blood pressure in dogs after 10 days [31] seems reasonable. When distributed over 10 days, the predicted median daily lead ingestion via trimmings would correspond to 5.3 – 27.5 mg/kg bw, which is far above the LOEL. Although the exact daily exposure is uncertain it still gives an idea of the level of exposure, to be compared to dose–response studies.

A daily oral dose of 2 mg lead /kg bw for 13 weeks reduced growth in puppies, and caused haematological signs of mild intoxication [30]. Pronounced lead intoxication was found in puppies fed 3.3 mg lead /kg bw/day for 12 weeks, in combination with a calcium and phosphorus deficient diet [24]. Thus, the predicted exposure in our study is found to be above these levels in many cases, and it is therefore likely that many dogs fed trimmings may experience toxic effects.

## Conclusions

Experimental data indicate that a daily dose of around 1 mg lead as lead acetate/kg body weight for ten days may be considered as a Lowest Observed Effect Level. Acute toxicity documentation from the Centers for Disease Control and Prevention indicates 300 mg/kg bw as the lowest single dose of lead acetate causing death in dogs after oral ingestion. Data gaps exist regarding the distribution of lead around the wound channel of lead-shot game, the intake of lead-contaminated meat by dogs, as well as the bioavailability of lead particles in dogs. However, available data suggest that dogs fed trimmings of lead-shot game may be affected by the amounts of lead present, and that even deadly exposure could occasionally occur. More research is needed to assess the exact consequences, if lead-based bullets are still to be used. Meanwhile, we recommend that trimmings close to the wound channel should be made inaccessible to dogs, as well as to other domestic or wild animals.

## Abbreviations

ALA, delta-aminolevulinic acid; ALA-D, aminolevulinic acid dehydratase; bw, body weight; LOEL, lowest observed effect level; Pb, lead
